# More management is needed to improve the effectiveness of artificial grassland in vegetation and soil restoration on the three-river headwaters region of China

**DOI:** 10.3389/fpls.2023.1152405

**Published:** 2023-04-21

**Authors:** Nengyu Wang, Jiayi Wan, Mingjun Ding, Hua Zhang, Shicheng Li, Linshan Liu, Yili Zhang

**Affiliations:** ^1^ School of Geography and Environment, Jiangxi Normal University, Nanchang, China; ^2^ Key Laboratory of Poyang Lake Wetland and Watershed Research, Ministry of Education, Jiangxi Normal University, Nanchang, China; ^3^ Department of Land Resource Management, School of Public Administration, China University of Geosciences, Wuhan, China; ^4^ Key Laboratory of Land Surface Pattern and Simulation, Institute of Geographic Science and Natural Resources Research, Chinese Academy of Sciences, Beijing, China; ^5^ College of Resources and Environment, University of Chinese Academy of Sciences, Beijing, China

**Keywords:** degraded alpine grassland, artificial grassland, plant-soil interaction, restoration management, partial least squares path models

## Abstract

Establishing an artificial grassland is a common measure employed to restore heavily degraded alpine grasslands for regional sustainability. The Three-River Headwaters Region in China has significant areas of black-soil-type grassland which is typified by heavy degradation; nearly 35% of the grassland regions in the Three-River Headwaters Region has degraded into this type. There are different plant community types of black-soil-type grasslands, however, it is not clear which restoration measures should be adopted for different kinds of black-soil-type grasslands. Here, we investigate the plant community characteristics and soil physicochemical properties of artificial grasslands, two types of black-soil-type grasslands, and native undegraded grassland in the Three-River Headwaters Region, then analyzed the direct and indirect interactions between the plant and soil properties by partial least squares path models (PLS-PM). Our results revealed that establishing artificial grassland significantly increased aboveground biomass and plant community coverage, and also decreased plant species richness and diversity and soil water content, soil organic carbon and total nitrogen in the 0-10 cm soil layer as compared with black-soil-type grasslands. Plant community diversity had a positive effect on plant community productivity, soil nutrient, and soil water content in native undegraded grassland. These results suggest that more management interventions are needed after establishing an artificial grassland, such as reducing dominant species in two types of black-soil-type grasslands, water regulation in the *A. frigida*-dominated meadow, diversifying plant species (i.e., Gramineae and sedges), and fertilizer addition.

## 1 Introduction

The Three-River Headwaters Region is located in the hinterland of the Qinghai-Tibetan Plateau, which is the source region of the Yangtze River, the Yellow River, and the Lancang (Mekong) River, known as “Asia’s water tower” (Mao et al., 2016). It is important for the ecological security of China and the countries surrounding the Qinghai-Tibetan Plateau (Shao et al., 2017). The alpine grassland biome is the main ecosystem of this area, which provides important ecosystem functions and services, such as climatic regulation (Liu et al., 2018; Dai et al., 2021), biodiversity conservation (Dong et al., 2020), soil erosion prevention (Wang et al., 2016) and habitat for both grazing livestock and wildlife (Liu et al., 2017; Lu et al., 2017; Liu et al., 2021), as well as the carbon sequestration (Feng et al., 2010; Chen X. et al., 2021). However, the Three-River Headwaters Region has experienced grassland degradation because of its fragile ecosystem, climate, and human influence (Guo et al., 2019; Wu et al., 2021). Nearly 35% of the grassland area has been heavily degraded into black-soil-type grassland, which is typified by bare land with no plants in the cold season and covered with forbs or poisonous plants in the warm season (Ma et al., 2002; Shang and Long, 2007; Dong et al., 2018). This has reduced plant coverage, species biodiversity, soil nutrient availability (Wen et al., 2013; Peng et al., 2018), and regional ecosystem stability (Dong et al., 2020; Yang and Sun, 2021). Furthermore, grassland degradation has an increasingly negative impact on regional security and social sustainability (Harris, 2010).

The restoration of the black-soil-type grassland is of vital importance and has received considerable study e.g., Wu et al., 2010; Dong et al., 2013; Wen et al., 2018). Approaches such as fencing enclosures (Chen X. et al., 2021; Du and Gao, 2021), application of fertilizers (Luo et al., 2017), or seeding (Shang et al., 2008) were conducted to restore degraded grasslands. However, none of these measures had significant positive effects on black-soil-type grasslands, which indicated that favorable natural restoration approaches were difficult for its rehabilitation (Shang and Long, 2007). One recent attempt to overcome this issue employed establishing Elymus nutans artificial grassland in black-soil-type grasslands (Feng et al., 2010; Wen et al., 2018). Some researches revealed that artificial grassland could be used as an effective restoration approach to improve productivity and regulate community and soil properties in black-soil-type degraded grasslands (Wu et al., 2010; Gao et al., 2019).

Many studies have analyzed the restoration effect of artificial grasslands by examining the effects on soil nutrients or vegetation characteristics at distinct intervals after the recovery work [e.g., 4-year, 6-year or 9-year (Wu et al., 2010; Gao et al., 2019)], and those results suggested an artificial grassland in black-soil-type grasslands requires a long-term for recovery (~ 16-18 years). However, there are different types of plant communities in black-soil-type grassland (Dong et al., 2010), but few studies have focused on the effect of rebuilding artificial grasslands as compared with different kinds of black-soil-type grasslands or healthy grasslands. It’s yet unclear whether the targeted restoration measures should be adopted in different kinds of black-soil-type grasslands. Furthermore, many studies have reported correlations between soil properties, such as soil moisture or nutrients, with alpine grassland properties, such as grassland aboveground biomass or biodiversity (Li et al., 2014; Fayiah et al., 2019; Faucon, 2020; Li et al., 2020; Xiao et al., 2022). Yet, little is known about the direct and indirect interaction between plant community characteristics and soil physicochemical properties, which is required for efficient and sustainable restoration practice in a degraded grassland ecosystem (e.g., ameliorate soil properties favoring autochthonous species) (Shen et al., 2015; Maiti and Ghosh, 2020; Peng et al., 2020).

Here, we investigate plant community characteristics and soil physicochemical properties in artificial *Elymus nutans* grasslands, two types of black-soil-type grasslands, and healthy *Kobresia* grassland. Our objectives are: (i) examine the differences in soil characteristics and plant communities among these grassland types, (ii) understand which targeted restoration measures (if any) should be adopted for different types of black-soil-type grasslands. The results of this study may help guide future grassland restoration programs in the Three-River Headwaters Region or other regions that face similar issues.

## 2 Materials and methods

### 2.1 Study sites

**Figure 1:**
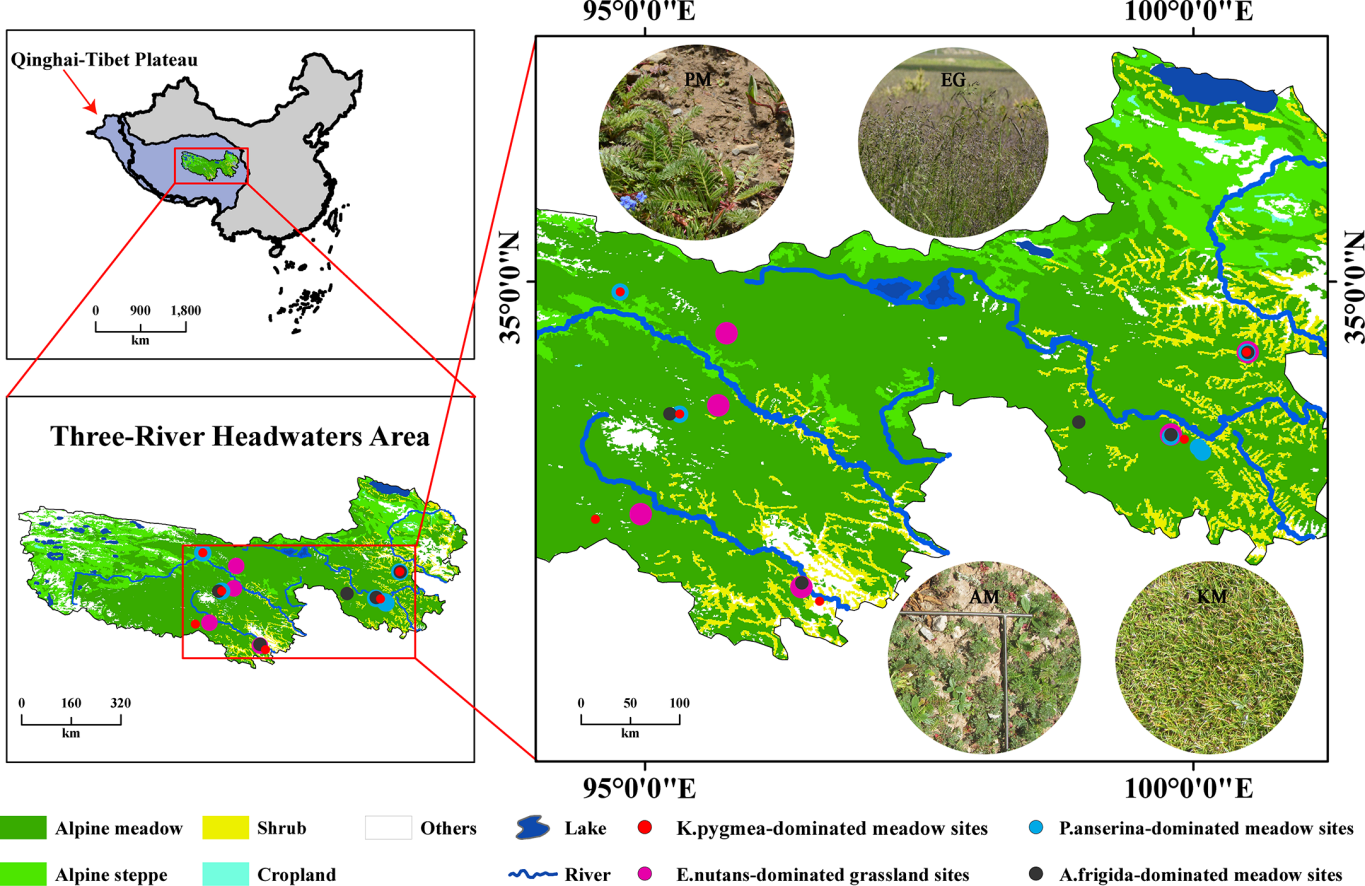
The distribution of vegetation types and sampling sites on the Three-River Headwaters Region. The vegetation map is based on a 1:1,000,000 scale vegetation distribution map of China (http://westdc.westgis.ac.cn). EG, *E.nutans*-dominated grassland; PM, *P.anserina*-dominated meadow; AM, *A.frigida*-dominated meadow; KM, *K.pygmea*-dominated meadow. Some sampling points are located relatively close to each other, resulting in overlapping symbols on the map.

The study area (31°45′N-39°19′N, 89°27′E-103°04′E) is located in the source region of the Yangtze River, the Yellow River, and the Lancang (Mekong) River (**Figure 1**). The altitude ranges from 2610 to 6950 m, with an average elevation of 4500m and many high mountains peaks. The site has a typical plateau continental climate with annual mean temperature ranges from -5.38 to 4.14°C, annual precipitation ranges from 262.2 to 772.8mm and annual evaporation rate ranges from 730 to1700 mm (Yi et al., 2012; Cao and Pan, 2014). The typical vegetation of the region are alpine meadows dominated by sedges and Gramineae, such as *Kobresia pygmaea*, *Kobresia capillifolia* and *Poa annua*. The soil is defined as alpine meadow in the Chinese Soil Classification System (Shi et al., 2006).

### 2.2 Field sampling design

Field sampling was conducted from July through August in 2019 and 2020 at the peak of the growing season to minimize differences due to the time of year. The sites were chosen to represent true replications of *K.pygmea*-dominated meadows (KM), *P.anserina*-dominated meadows (PM), *E.nutans*-dominated grasslands (EG), and *A.frigida*-dominated meadows (AM). KM represents healthy grassland, PM and AM are two kinds of black-soil-type grasslands, and EG is the artificial grassland (**Table 1**). We established six sites for each kind of grassland. At each site, a 10m×10m plot was randomly chosen. Within each plot, three 0.5m×0.5m quadrats were placed to survey vegetation and soil. In total, 72 quadrats (24 sites × 3 quadrats) were sampled, and the geographical coordinates were also recorded for each plot. Black-soil-type grasslands tend to occur near artificial grasslands, therefore, sometimes these sampling sites would be set up in the same area.

**Table 1:** Characteristics of sampling sites in the Three-River Headwaters Region.

	Grassland type	Abbreviation	Altitude(m)	Main plant species
Healthy grassland	*K.pygmea*-dominated meadow	KM	4318	*Kobresia pygmaea, Poa annua*, *Carex alatauensis, Carex myosuroides*
Black-soil-type grasslands	*P.anserina*-dominated meadow	PM	4208	*Potentilla anserina, Knorringia sibirica*, *Lagotis brachystachya, Microula sikkimensis*
	*A.frigida*-dominated meadow	AM	4118	*Artemisia frigida, Ajuga lupulina*, *Elsholtzia densa*
Artificial grassland	*E.nutans*-dominated grassland	EG	4130	*Elymus nutans, Poa annua*

### 2.3 Plant community survey

We investigated plant community characteristics, including species identity, height, coverage, abundance and aboveground biomass of each species in each quadrat. Plant coverage was represented by the ratio of the shady area of a specific species to the total area of a quadrat. The plant species were clipped and then put into an envelope for each quadrat. We determined the aboveground biomass for every quadrat by weighing the plants after drying at 65°C to a constant weight. We calculated the Gleason index (G), Shannon-Wiener index (H′), Simpson index (D) and Pielou index (J) to characterize the richness, diversity and evenness of plant community:


(1) Pi=(RC+RA+RH)/3

(2) G=S/InA

(3) $$H^{\prime }=-\sum^{\;}\mathrm{PiInPi}$$

(4) $$D=1-\sum Pi^{2}$$

(5) $$J=H^{\prime }/\mathrm{InS}$$

where *Pi* is the important value of the species in the plant community site, *RC* is the relative coverage, *RA* is the relative abundance, and *RH* is the relative height. *S* is the sum of the species in the site and *A* is the area of the site.

### 2.4 Soil physicochemical properties’ measurement

We collected topsoil (0-5 cm) and subsoil (5-10 cm) samples from the plot after the plant community survey. Oven-drying was used to measure soil bulk density (BD) and soil water content (SW) through drying the soil sample of the steel cutting rings. Soil pH and electrical conductivity (EC) were measured from a soil water ratio of 1:2.5 with a pH meter and conductivity meter. Soil organic carbon (SOC) was determined using the dichromate oxidation method. Total nitrogen (TN) was analyzed by the Kjeldahl method. Total potassium (TK) and total phosphorus (TP) were measured by flame photometer and molybdenum antimony resistance colorimetry after wet digestion with H_2_SO_4_ and HCLO_4_. The soil particle size composition was measured by the laser scattering particle size distribution analyzer, and we classified soil as clay, silt and sand by international particle size standards.

### 2.5 Analysis of the plant-soil interaction

The Shapiro-Wilk normality test and Bartlett’s test of homogeneity were performed to check for normality and equal variance among groups. The plant community characteristics of four groups were non-normal data and all showed variance heterogeneity. Thus, we compared the vegetation parameters between four groups with the Mann-Whitney U test by using the wilcox.test function in R. Spearman correlation coefficients were used to characterize the relationship between the soil properties and the plant community. To screen the important soil properties that influenced the plant community, we performed the random forest model with R-package “linkET”.

We constructed a partial least squares path models (PLS-PM) that provide a comprehensive view of a system by modeling multiple relationships between its components to better integrate the interaction among plant community characteristics and soil physicochemical properties. In the PLS-PM framework, a latent variable is viewed as a concept and is linked to a set of measurements. Our latent variables included soil physical properties, soil nutrient content, plant characteristics, and plant diversity. We identified these latent variables by choosing the important soil properties based on the results of the random forest model, for example, soil nutrients including SOC and TN. PLS-PM was performed using the “plspm” R-package. All statistical analyses were conducted using R version 4.1.2 unless noted otherwise.

## 3 Results

### 3.1 Vegetation parameters in different plant community grasslands

**Figure 2:**
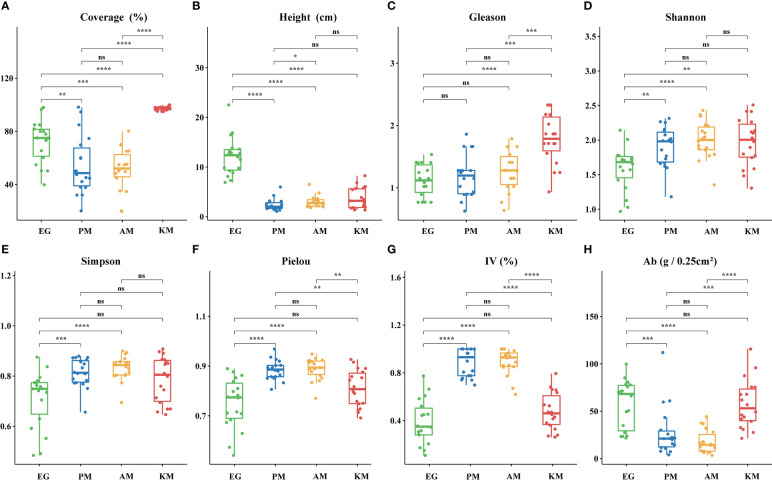
Boxplot of vegetation parameters. **(A)** Coverage of plant community; **(B)** Height of plant community; **(C)** Gleason index; **(D)** Shannon-Wiener index; **(E)** Simpson index; **(F)** Pielou index; **(G)** Important value of forbs; **(H)** Aboveground biomass. Significant *P* values were shown in boxplot. * means adjusted *P* values ≤ 0.05, ** means adjusted *P* values ≤ 0.01, *** means adjusted *P* values ≤ 0.001 and **** means *P* ≤ 0.0001, if not indicated, means adjusted *P* values > 0.05. EG, *E.nutans*-dominated grassland; PM, *P.anserina*-dominated meadow; AM, *A.frigida*-dominated meadow; KM, *K.pygmea*-dominated meadow.

The four kinds of plant community grasslands showed different plant community characteristics (**Figure 2**). The coverage and Gleason index were significantly higher in KM as compared with the other three kinds of plant community grasslands (*P* ≤ 0.001, **Figures 2A, C**), while PM and AM exhibited no significant differences (**Figures 2A, C**). The average height of EG was the highest (*P* ≤ 0.0001, **Figure 2B**), but there were no significant differences in the other three groups. The important value of forbs and Pielou evenness index in PM and AM were higher than that in EG and KM (*P* ≤ 0.0001, **Figure 2G**). Conversely, the aboveground biomass of EG and KM was significantly greater than PM and AM (**Figure 2H**). The plant diversity index (Shannon-Wiener and Simpson) was similar among PM, AM, and KM, and the lowest diversity index was recorded in EG (**Figures 2D, E**). In addition, the Pielou index of PM and AM was significantly higher than KM and EG (*P* ≤ 0.01, **Figure 2F**).

### 3.2 Soil physicochemical properties in different plant community grasslands

**Figure 3:**
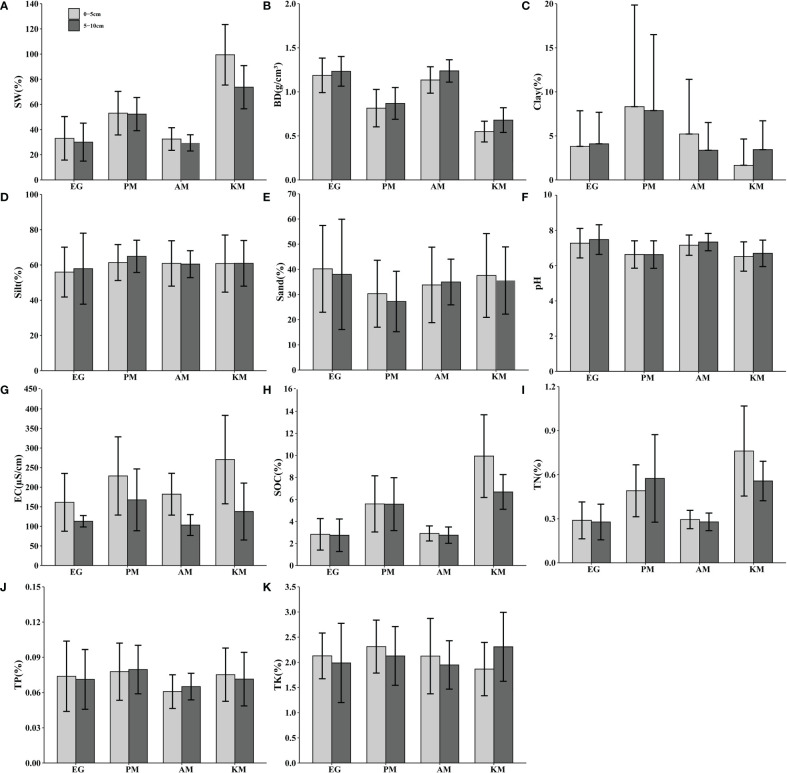
Soil physicochemical properties of plant communities in different alpine grasslands. Error bars indicate standard error. **(A)** SW(%), soil water content; **(B)** BD(g/cm^3^), bulk density; **(C)** Clay(%), clay percentage; **(D)** Silt(%), silt percentage; **(E)** Sand(%),sand percentage; **(F)** pH, Potential of Hydrogen; **(G)** EC(μS/cm), Electrical Conductivity; **(H)** SOC(%), Soil Organic Carbon; **(I)** TN(%), Total Nitrogen; **(J)** TP(%), Total Phosphorous; **(K)** TK(%), Total Potassium; EG, *E.nutans*-dominated grassland; PM, *P.anserina*-dominated meadow; AM, *A.frigida*-dominated meadow; KM, *K.pygmea*-dominated meadow.

The soil water content, soil organic carbon and total nitrogen of EG and AM was less on average compared with the other two kinds of grasslands, especially the surface layer soil (0-5cm) of KM (**Figures 3A, H, I**). Correspondingly, KM had the lowest soil bulk density (0.55g/cm^3^ in 0-5cm soil layer, 0.68g/cm^3^ in 5-10cm soil layer), and PM followed (**Figure 3B**). In addition, silt content in four kinds of alpine grassland was the highest, followed by sand and clay content (**Figures 3C–E**). The differences in silt content among these alpine grasslands were marginal, but the clay content in PM was significantly higher than the other three kinds of grasslands (**Figures 3C, D**). Soil pH in EG (7.28 in 0-5cm soil layer, 7.48 in 5-10cm soil layer) and AM (7.16 in 0-5cm soil layer, 7.33 in 5-10cm soil layer) was higher than in KM and PM, whereas the electrical conductivity in EG and AM was lower than KM and PM (**Figures 3F, G**). The electrical conductivity of the 0-5cm soil layers was higher than the 5-10cm soil layers among all kinds of grassland (**Figure 3G**). Total phosphorous was lowest in AM but showed no differences in the other three kinds of grassland (**Figure 3J**). Furthermore, total potassium of the 0-5cm soil layers in KM (0.075%) was significantly higher than the 5-10cm soil layers, but it was opposed to the other three kinds of grassland (**Figure 3K**).

### 3.3 Plant-soil interaction

**Figure 4:**
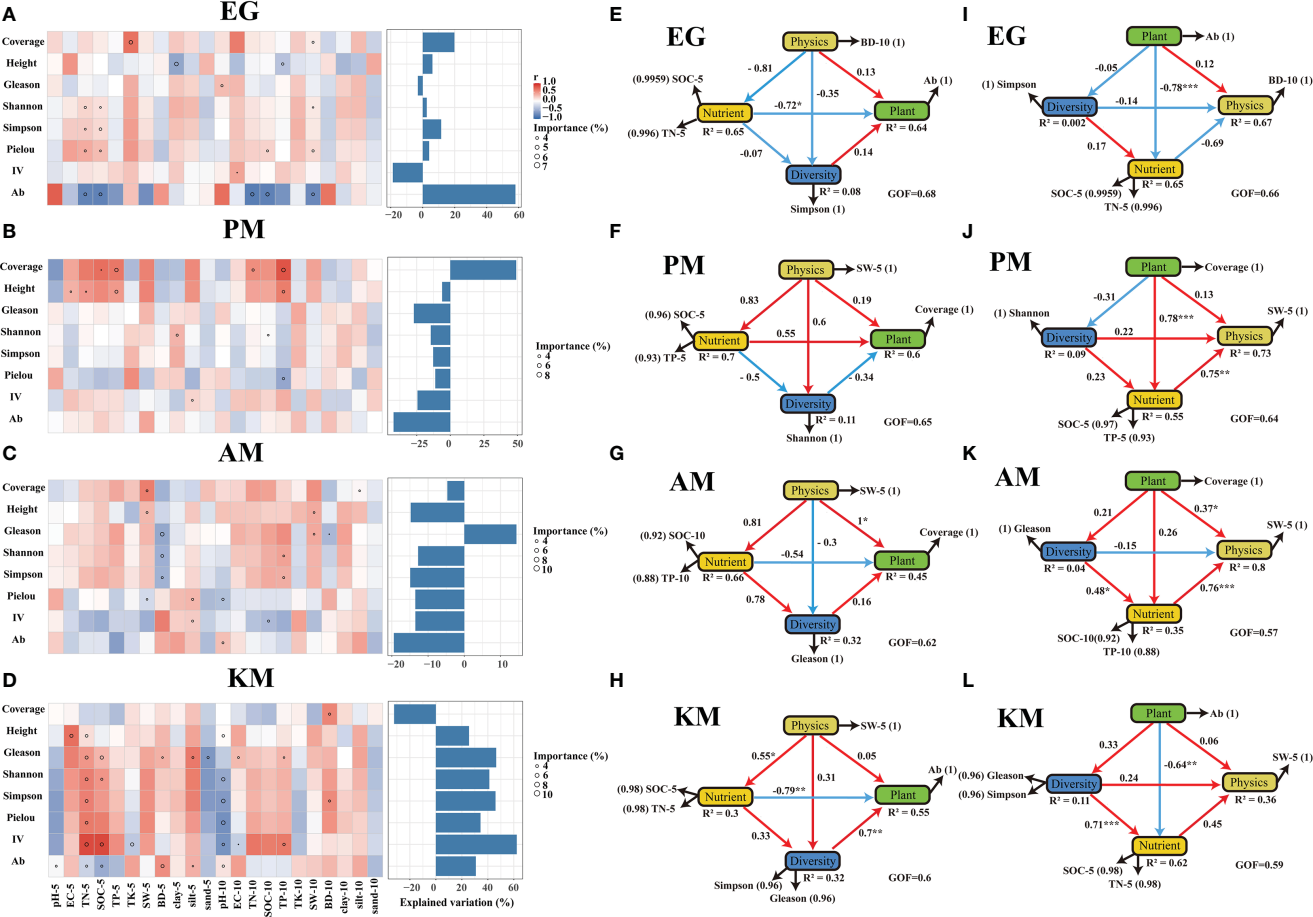
The correlations and interactions between plant communities and soil physicochemical properties. **(A–D)** Contributions of soil properties to the vegetation parameters based on correlation and a random forest model. The circle size represents the importance of the variables (percentage of increase of mean square error calculated via random forest model). Colors represent Spearman correlations. Diagrams **(E–L)** of partial least squares path models (PLS-PM) describing the interactions among the soil physicochemical properties and plant community characteristics. Each box represents a latent variable, the manifest variables are the indexes beside the latent variables, and the value in the brackets represent the loading values. Arrows connecting latent variables indicate inner model paths, with blue and red indicating negative and positive effects, respectively. R^2^ denotes the proportion of variance explained. * means adjusted P values ≤ 0.05, ** means adjusted P values 175 ≤ 0.01, *** means adjusted P values ≤ 0.001.

The important value of forbs was positively correlated with SOC, TN, and TP in the 0-5cm and 5-10cm soil layers but was negatively correlated with pH, TK, and sand percentage in the 0-5cm and 5-10cm soil layers in KM. This was more pronounced in the surface soil layer of 0-5 cm (**Figure 4D**), but was only positively correlated with EC in the 5-10cm soil layer of EG (**Figure 4A**). Aboveground biomass was negatively correlated with TN, SOC, TP, and SW in the 0-10cm soil layer and positively correlated with pH, and BD in the 0-10cm soil layer of EG (**Figure 4A**); No soil physicochemical properties were significantly correlated with the aboveground biomass of PM (**Figure 4B**). In addition, BD of the 0-5cm soil layer was negatively correlated with the coverage, richness, and diversity of plant community but was positively correlated with important value of forbs, aboveground biomass, and the Pielou index for AM (**Figure 4C**). Furthermore, the relationship between plant community characteristics and physicochemical properties was tighter in KM than the other three kinds of grassland, especially plant diversity and soil nutrient availability.

PLS-PM seems to explained a majority of the soil nutrient variability in EG and KM (R^2 =^ 0.65 and 0.62 resp., **Figures 4E, L**) and the physical properties (SW at 0-5cm soil layer) were well explained in PM and AM (R-square =0.73 and 0.8 resp., **Figures 4J, K**). There was a significant direct negative interaction between soil nutrients and aboveground biomass of plant communities in EG and KM (**Figures 4E, H, I, L**), meanwhile, the effect of plant diversity on plant community aboveground biomass was significantly positive (path coefficient = 0.7, *P* values ≤ 0.01, **Figure 4H**) in KM but had only a marginal positive effect (path coefficient = 0.14, **Figure 4E**) in EG. In addition, the effect of plant community coverage on soil nutrient availability were both positive in PM and AM (path coefficient = 0.78 and 0.26 resp., **Figures 4J, K**), and the effect of soil nutrients on plant community coverage was negative in AM (path coefficient = -0.54, **Figure 4G**) but positive in PM (path coefficient = 0.55, **Figure 4G**). Moreover, the direct effects of plant community diversity on soil nutrients were positive among four types of plant community grasslands (path coefficient = 0.17, 0.23, 0.48 and 0.71 resp., **Figures 4I–L**), in which the effect was highly significant (P values ≤ 0.001, **Figure 4L**) in the KM and marginal in EG (**Figure 4I**). However, the direct effects of soil nutrients on plant community diversity were negative in EG and PM (path coefficient = -0.07 and -0.5 resp., **Figures 4E, F**) but positive in AM and KM (path coefficient = 0.78 and 0.33 resp., **Figures 4G, H**).

The interactions between soil physical properties and plant community characteristics were significant in AM (path coefficient = 1 and 0.37 resp., *P* values ≤ 0.05, **Figures 4G, K**) but marginal among EG, PM, and KM (path coefficient<0.2, **Figures 4E–L**). However, the direct interactions between soil physical properties and plant community diversity were both negative in EG (path coefficient = -0.35 and -0.14 resp., **Figures 4E, I**) and AM (path coefficient = -0.3 and -0.15 resp., **Figures 4G, K**) but positive in PM (path coefficient = 0.6 and 0.22 resp., **Figures 4F, J**) and KM (path coefficient = 0.31 and 0.24 resp., **Figures 4H, L**). In addition, certain soil physical properties (SW at 0-5cm soil layer) had an indirect effect (path coefficient = -0.41 and 0.64 resp., **Table 2**) on plant community diversity through the effect on soil nutrients in PM and AM. Moreover, there was an indirect positive effect of plant community diversity on the physical properties of soil through the beneficial effect of soil nutrients in PM, AM and KM (path coefficient = 0.18,0.7 and 0.32 resp., **Table 2**). In general, the effect of plant on soil was greater than the effect of soil on plant in EG, PM and KM and the plant-soil interaction was significant greater in the healthy *Kobresia* meadow than the artificial *Elymus* nutans grassland and the two types of black-soil-type grasslands (**Figure 4**).

**Table 2:** Direct and indirect path effects of latent variables based on partial least squares path modeling (PLS-PM).

Path	EG		PM		AM		KM	
	Direct	Indirect	Direct	Indirect	Direct	Indirect	Direct	Indirect
Physics -> Nutrient	-0.81	0	0.83	0	0.81	0	0.55	0
Nutrient -> Physics	-0.69	0	0.75	0	0.76	0	0.45	0
Physics -> Diversity	-0.35	0.06	0.6	-0.41	-0.3	0.64	0.31	0.18
Diversity -> Physics	-0.14	-0.12	0.22	0.18	-0.15	0.37	0.24	0.32
Nutrient -> Diversity	-0.07	0	-0.5	0	0.78	0	0.33	0
Diversity -> Nutrient	0.17	0	0.23	0	0.48	0	0.71	0
Nutrient -> Plant	-0.72	-0.01	0.55	0.17	-0.54	0.13	-0.79	0.23
Plant -> Nutrient	-0.78	-0.01	0.78	-0.07	0.26	0.1	-0.64	0.24
Diversity -> Plant	0.14	0	-0.34	0	0.16	0	0.7	0
Plant -> Diversity	-0.05	0	-0.31	0	0.21	0	0.33	0
Plant -> Nutrient	-0.78	-0.01	0.78	-0.07	0.26	0.1	-0.64	0.24
Nutrient -> Plant	-0.72	-0.01	0.55	0.17	-0.54	0.13	-0.79	0.23

## 4 Discussion

The establishment of artificial grasslands is a common practice to restore vegetation and soil in many extremely degraded grasslands in alpine areas (Li et al., 2013). Our investigation in the Three-River Headwaters Region demonstrated that artificial grassland increased aboveground biomass but decreased plant species richness and diversity, which is consistent with previous studies on artificial grasslands of alpine grassland ecosystem (Shang et al., 2008; Feng et al., 2010; Wu et al., 2010). This may be attributed to the fact that *Elymus nutans* have greater competitive ability and increase in abundance than forbs. On one hand, the *Elymus nutans* is taller than other native species and produces shading effects, which may limit the growth of short species because of their advantage for light (Wu et al., 2010; Peng et al., 2023). On other hand, the *Elymus nutans* generally have deeper roots than other plant species, which may have enabled the plant community to acquire more water and thus increase primary production (Liu et al., 2018).

We also found that the establishment of artificial grassland did not improve the soil water, soil organic carbon, or total nitrogen, which suggests a lower water-holding capacity and increased loss of soil nutrients. This is inconsistent with the findings of Wu et al. (2010), who reported that soil nutrient properties all significantly increased in artificial grassland. However, Dong et al. (2012) reported that the establishment of artificial grassland did not restore soil quality or nutrient stocks in the headwaters of the Yellow River. And different grassland types may respond differently to a given restoration measure. Previous studies reported that there was a reoccurrence of black-soil-type grasslands in artificial grassland projects (Zhang et al., 2018), which suggests that the soil quality of artificial grassland struggles to maintain the sustainable growth of *Elymus nutans*. Most of the artificial grassland is seriously lacking in scientific management after establishment (Shang et al., 2018; Dong et al., 2020). Our study indicated that the soil water and nutrient content of artificial grasslands are much worse than that of natural meadows (**Figure 3**). Therefore, protective measures should be taken to alleviate soil erosion and the loss of soil nutrients after the establishment of artificial grassland. Mulching the non-woven materials in the artificial grassland may significantly boost seed germination rate and reduce soil erosion (Li et al., 2019; Chen B. J. et al., 2021). Further studies are required to explore the soil quality resilience of the artificial grassland across the restoration time.

We found that the restoration efficiency of artificial grasslands is significantly different compared with the two kinds of black-soil-type grasslands. The soil nutrient and soil water content in the *P.anserina*-dominated meadow were significantly higher than *E.nutans*-dominated grassland and *A.frigida*-dominated meadow (**Figure 3**), implying the restoration efficiency of the artificial grassland in *P.anserina*-dominated meadow was lower than the *A.frigida*-dominated meadow. This result may reflect plant community could steer grassland vegetation via the effect of soil (Peng et al., 2020; Heinen et al., 2020; Xu et al., 2022). For instance, *Potentilla anserina* in *P.anserina*-dominated meadow is the perennial stoloniferous clonal plant that has a strong reproductive capacity at the low tropic level (Saikkonen et al., 1998). Due to its fast-growing creeping stem (Li, 2004), the coverage of the *P.anserina*-dominated meadow is higher in general, which is beneficial to soil water conservation. In addition, the development of the root system of *Potentilla anserina* (Kang, 2007) indicates that the soil organic matter content is relatively high. However, *Artemisia frigida* in the *A.frigida*-dominated meadow adapts to soil with low nutrients (Yan et al., 2016) and also has strong allelopathy (Li et al., 2011). The allelochemicals of *A.frigida* may be produced in leaves and roots, from which they can be released into the soil to inhibit the germination and growth of other plant species (Mutlu et al., 2009). Furthermore, we found that the negative effect (path coefficient = -0.54) of soil nutrient on plant community coverage was larger than the positive effect (path coefficient = 0.26) of plant community coverage on soil nutrient in the *A.frigida*-dominated meadow (**Figures 4G, K**). It suggested that the plant species might have a high efficiency of nutrient utilization in low nutrient soil (**Figures 3H, I**) and contribute little to fertile soil which may be due to their allelochemicals. This implied that *A.frigida* and other weeds should be eradicated, and fertilizer addition should be considered when restoring the *A.frigida*-dominated meadow. More knowledge is required on the plant growth patterns in different kinds of black-soil-type grasslands and the plant-soil interactions of these grasslands.

## 5 Conclusions

The establishment of artificial grassland improved vegetation productivity and plant community coverage when compared to two kinds of black-soil-type grasslands. However, restoration could not fully achieve the recovery of plant community diversity, soil nutrient content, or soil water content, even though the soil nutrient content and water were lower than in *P.anserina*-dominated meadows. In general, artificial grasslands did not restore the two types of black-soil-type grasslands to the level seen for native undegraded grassland. This could indicate that management interventions such as fertilizer addition should be implemented after establishing the artificial grassland. In addition, our results show that the soil nutrient content in the *P.anserina*-dominated meadows was higher compared with *A.frigida*-dominated meadows. We hypothesize that the dominant species in the *A.frigida*-dominated meadows has strong allelopathy which could inhibit the growth of plant species and lead to nutrient-poor soil. Moreover, soil water content had a significant positive influence on plant community and soil nutrient content in the *A.frigida*-dominated meadows. Consequently, the forbs should be reduced, and water and fertilizer should be added during the restoration of *A.frigida*-dominated meadows. Furthermore, plant community diversity had a positive effect on plant community productivity, soil nutrient content, and soil water in native undegraded grassland, which indicated that plant diversity was beneficial for the stability of the plant community. Therefore, more plant species such as Gramineae and sedges should be planted in artificial grasslands. Our findings may be used in the restoration of *P.anserina*-dominated meadows and *A.frigida*-dominated meadows and in the management of artificial grasslands.

## Data availability statement

The raw data supporting the conclusions of this article will be made available by the authors, without undue reservation.

## Author contributions

NW, MD, and HZ contributed to conception and design of the study. NW, JW, MD, HZ organized the database and performed the statistical analysis. NW wrote the first draft the manuscript. NW, JW, MD, HZ, SL, LL and YZ reviewed and revised the manuscript. All authors contributed to the article and approved the submitted version.
